# An Analysis of Changes in the Harmfulness of the Bottle Packaging Process Depending on the Type of Heat-Shrinkable Film

**DOI:** 10.3390/ma17164115

**Published:** 2024-08-20

**Authors:** Patrycja Walichnowska, Weronika Kruszelnicka, Adam Mazurkiewicz, Zbigniew Kłos, Anna Rudawska, Michał Bembenek

**Affiliations:** 1 Faculty of Mechanical Engineering, Bydgoszcz University of Science and Technology, Al. Prof. S. Kaliskiego 7, 85-796 Bydgoszcz, Poland; weronika.kruszelnicka@pbs.edu.pl (W.K.); adam.mazurkiewicz@pbs.edu.pl (A.M.); 2 Institute of Machines and Motor Vehicles, Faculty of Transport Engineering, Poznan University of Technology, 60-965 Poznan, Poland; zbigniew.klos@put.poznan.pl; 3 Department of Production Engineering, Faculty of Mechanical Engineering, Lublin University of Technology, 36 Nadbystrzycka Str., 20-618 Lublin, Poland; a.rudawska@pollub.pl; 4 Department of Manufacturing Systems, Faculty of Mechanical Engineering and Robotics, AGH University of Krakow, 30-059 Krakow, Poland

**Keywords:** environmental impact, heat-shrinkable film, recycled plastics, life cycle assessment (LCA)

## Abstract

This article shows an analysis of selected stages of a machine’s life cycle environmental impact in the specific case of machines that package bottles in thermo-shrinkable film. As part of this analysis, laboratory tests were carried out to compare the performance properties of polyethylene films (with and without recycled material). Then, a life cycle assessment (LCA) was carried out within the specified system boundaries using the SimaPro program. Using the ReCiPe 2016 method, differences in the impact of the mass bottle packaging process on the categories human health, ecosystems and resources were determined depending on the shrink film used in the process. These tests showed that the tested batch of film with the addition of recyclates has similar functional properties to traditional ones and can therefore be used in the mass packaging process. The environmental analysis showed that changing the type of film to film with the addition of recyclates results in an almost 70% reduction in the potential negative impact of the process in terms of damage to health and ecosystems, and by 85% in terms of resources.

## 1. Introduction

One of the main problems of the modern times is the production of too large amounts of harmful substances that exacerbate global warming, the depletion of the ozone layer and excessive amounts of landfill waste [1]. Manufacturing processes have an important role in the emission of pollution into the atmosphere. Work on reducing the harmfulness of these processes applies to every manufacturing company. The desired effect can be achieved through investments in companies, i.e., the purchase of a new packaging machine or reducing financial outlays by introducing changes in the smoothness of a process [1,2,3]. Reducing the harmfulness of the process of the mass packaging of bottles in heat-shrinkable film is associated with obtaining useful and useless products of the process, which are packs of bottles. Increasing the amount of useful products contributes to increasing the efficiency of the process, but also to reducing the demand for energy and to reducing the amount of waste generated in the mass packaging process, i.e., ultimately to minimizing the harmful impact on the technical system [4,5,6]. The process of the bulk packaging of bottles, like any process, also has a significant impact on the environment. Therefore, it is considered appropriate to search for both a highly efficient and a less harmful variant of this process.

The environmental impact of mixed-material packaging has been the subject of much work since the early development of life cycle assessment (LCA) methodologies, and there is now an extensive body of research in this area. The literature contains many considerations regarding polyethylene and its environmental impacts. Yao et al. [7] described the properties of polyethylene and its impact on the environment. In terms of analysis, the authors proposed a direction for testing the toxicity of this polymer. The authors said that plastic materials, including polyethylene, improve comfort in everyday life, but they are dangerous for the environment and threaten human health. Harding et al. [8] conducted an LCA analysis of the production of a biodegradable polymer (PHB), which may be an alternative to traditional polyethylene. In their analysis, the authors checked the CO_2_ generation of this process and all the main impact categories. The obtained results were compared with similar tests on traditional polyethylene (PE), which showed that PHB has a lower potential impact in most impact categories. Only in the case of acidification and eutrophication impacts was this impact greater. Brock and Williams [9], in their research, conducted an analysis to determine whether there is any beverage packaging that has a lower impact on the environment than a plastic bottle. In their analysis, the authors compared glass bottles, aluminum cans, high-density polyethylene (HDPE) bottles, Tetra Pak bottles, polyethylene terephthalate (PET) bottles and milk cartons. The research showed that 100% recycled aluminum cans would be the least impactful option for pressurized drinks, Tetra Pak bottles for fruit drinks and cartons for milk. Martín-Lara et al. [10] analyzed the post-consumer recycling process of polyethylene films. Their research showed that the washing stage has the greatest impact on the environment, mainly due to electricity consumption. Their research showed that the entire mechanical recycling process causes damage, mainly to human health, which accounts for as much as 93.4% of the total impact of the process. Tan et al. [11] conducted a comparative LCA analysis of mulching films that differed in thickness, materials and end-of-life options. The authors showed that biodegradable film is more environmentally friendly than polyethylene film. They indicated that potential improvements in greener product design by increasing the use of bio-based materials should be considered. The available literature contains many studies on the life cycle analysis of polyethylene packaging which is used in various industries [12,13,14,15,16,17,18,19]. Most research focuses on the analysis of the life cycle of various types of polymer films, including polyethylene and recycled and biodegradable films [20,21,22,23,24,25]. There is still no way to determine how changing film packaging with the addition of recycling affects the harmfulness of the mass packaging of bottles. The main aim of the article is to compare the harmfulness of the process in three impact categories, i.e., human health, ecosystems and resources, depending on the type of packaging material used. A comparison of the functional properties of the film and the harmfulness of the mass bottle packaging process depending on the type of film used is the subject of this article. This work includes an analysis of the impact of changing the type of film on the harmfulness of the bulk bottle packaging process in three categories of damage (human health, ecosystems, resources). The main goal was to analyze the harmfulness of the tested process in two variants using the LCA method. The boundaries of the system included five stages of the packaging process: supplying products, formatting products, wrapping a group of bottles with film, shrinking the film and cooling the packs. Further stages, including the post-consumer management of used products, were excluded from this research. To be able to compare the examined processes, 1000 packs were assumed to be a functional unit. The analysis was carried out using the SimaPro 9.3 software. The obtained results were presented in the form of tables and graphs, which were discussed and interpreted accordingly. Laboratory tests were also carried out to compare the operational properties of 0rLDPE/100LDPE and 50rLDPE/50LDPE films. In the tests of tensile strength, the tear strength and shrinkage of the films were compared. The novelty and added value of this study lie in the determination of the impact of changing the film used in the mass packaging of bottles on the harmfulness of this process. We carried out determination of environmental impacts using the LCA method and a comparison of the material properties of film samples in light of the impact of the tested process on the environment.

## 2. Materials and Methods

**Figure 1:**
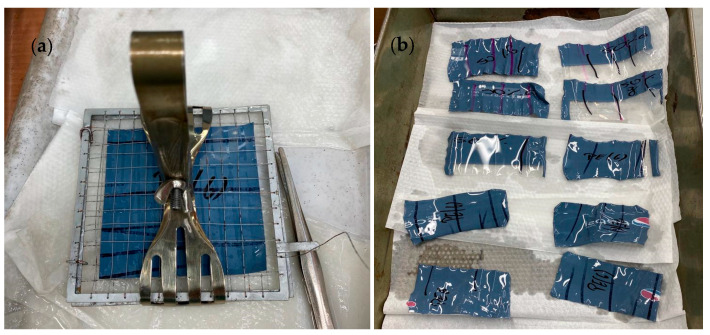
Film samples before (**a**) and after (**b**) the free-shrinkage test, the lines on the films indicated the longitudinal direction (own study).

One of the goals of this research was to determine the operational properties of the film. The samples of the film had thicknesses of 0.046 mm (0rLDPE/100LDPE) and 0.045 mm (0rLDPE/100LDPE). The samples were obtained from a company producing heat-shrinkable films. The basic source of information about the mechanical properties of plastics is the static tensile test. It involves axial stretching of material samples with precisely defined standardized shapes in holders. In the case of easily deformable materials, Young’s modulus is of theoretical importance. The actual stresses corresponding to the deformations that may occur during operation differ significantly from those that could be expected based on the elastic modulus. When characterizing the functional properties of polymer materials, the modulus of elasticity is often not provided; however, in this study, stresses corresponding to individual values of relative elongation are determined. Therefore, in the tests carried out, the relative elongation at the yield point, at maximum stress and at the breaking of the analyzed films was determined [26,27]. To determine the mechanical properties under static tension, tests were carried out according to the PN-EN ISO 527-1:2020 [28] and PN-EN ISO 527-3:2019-01 [29] standards. In the first step, 15 mm wide film samples were cut in the machine and transverse directions. The tests were carried out on a TIRAtest 27025 machine, TIRA, Schalkau, Germany. To determine the shrinkage of the film, a linear shrinkage test was carried out, which involved determining the changes that occur in the plastic material under the influence of temperature. This method is used to assess the suitability of a given heat-shrinkable film made of various plastics for packaging purposes. Tests were performed in accordance with ASTM D2732 [30], a standard test method for the unconfined linear thermal shrinkage of films using a Memmert machine with a bowl along with silicone oil. To carry out the test, samples with dimensions of 100 × 100 mm were prepared, with the longitudinal direction marked on them. The samples, placed in a special holder (Figure 1), were immersed in silicone oil at a temperature of 140 °C for 10 s. It was important not to hold the samples above the oil pan before immersion because of the possibility of premature shrinkage or annealing of the film. After 10 s, the samples were removed and placed on a tray to cool and drain excess oil. After the test, the linear dimensions of the samples were measured and recorded. Linear free-shrinkage testing in the longitudinal direction was performed in accordance with ASTM D2732.

To determine the force needed to tear thin film samples at a set length from the cut under specific load conditions (1600 g), an Elmendorf test was carried out in accordance with the PN-EN ISO 6383-2:2005 standard using a Pro Tear Tearing Tester, Twing machine—Albert Instrument Co, New Berlin, NJ, USA [31].

To assess the change in the process impact, the Life Cycle Assessment method was used, which is used to determine the impact on the systemic environment of processes within established limits, i.e., “from entry to exit of the same organization” [32,33,34]. This research used the ReCiPe 2016 method, in which impacts are characterized at the midpoint and endpoint levels [35,36,37]. LCA analysis involves four stages that are closely related to each other:

- Goal and the Scope definition,
- Life cycle investment analysis,
- Life cycle impact assessment,
- Life cycle interpretation.

**Figure 2:**
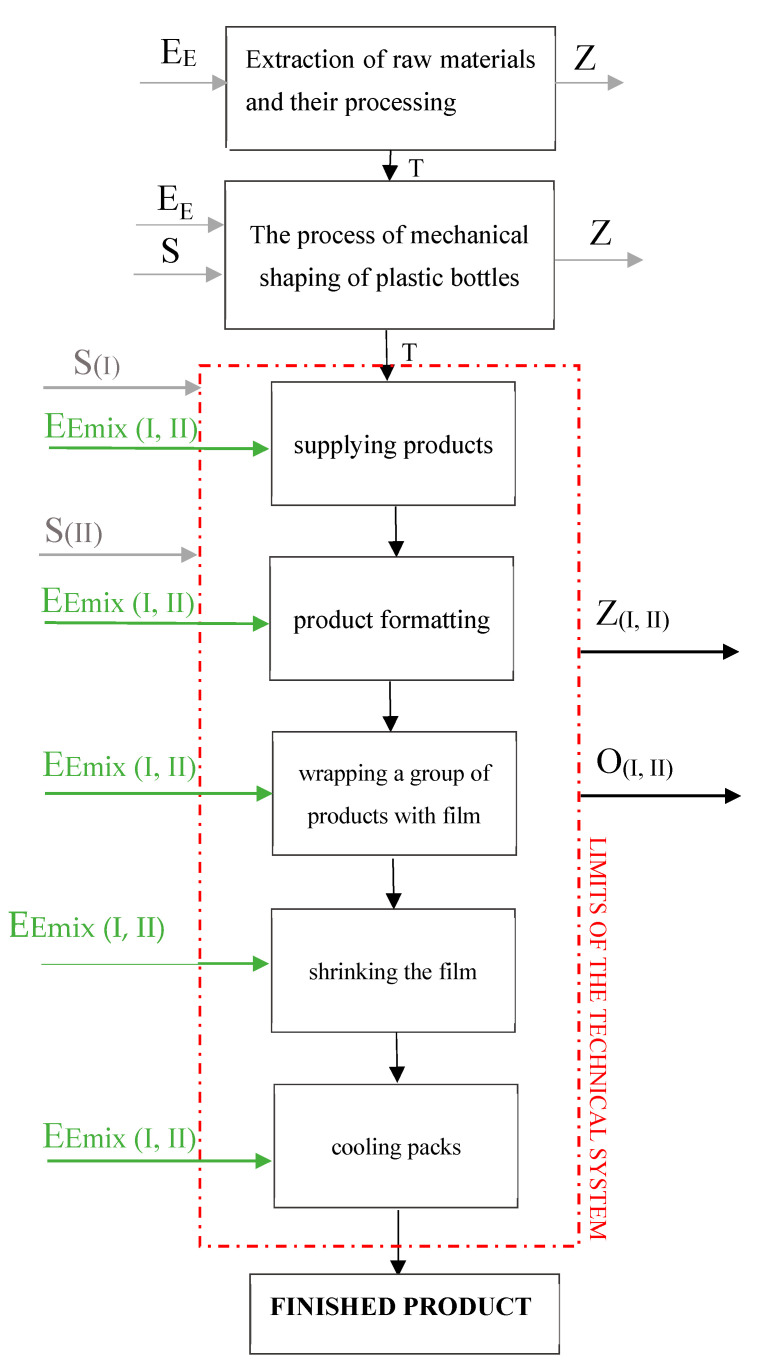
Diagram of process of mass packaging of bottles including input and output data: EE—electricity; S—raw materials; Z—pollution; T—transport; S_(I)_—raw materials as input data in process variants I; S_(II)_—raw materials as input data in process variants II; E_Emix(I, II)_—electricity in process variants I; Z_(I, II)_—pollution in form of greenhouse gas emissions (CO_2_, SO_2_, SF_6_, N_2_O); O_(I, II)_—waste in form of unusable packs (own study).

In the first step of LCA, the purpose and scope of the analysis are determined. The intended use of the research results must be established and this choice justified. It is important to determine whether the analysis being conducted is comparative in nature. In such a case, systems should be compared using the same functional unit, the same operational properties, system boundaries and data quality, and the same decision rules for assessing inputs and outputs [38,39]. Bearing in mind the specific purpose of this research, the LCA analysis compared the impacts of the mass packaging process in two different variants, which differed in the type of heat-shrinkable film used. Due to the fact that this is a comparative study, equivalence of the studied processes was ensured at the beginning. This involved adopting the same functional unit, system boundaries, data quality and decision rules regarding inputs and outputs for comparison, which made it possible to identify the most environmentally burdensome factor of the mass packaging process and provide indications for its further development. Based on the data collected from the mass packaging processes carried out in the company, a functional unit was established, i.e., 1000 packs produced by a given packaging machine. The boundaries of the adopted system include process stages such as supplying products, formatting products, wrapping a group of bottles with film, shrinking the film and cooling the packs (Figure 2). The research excluded the stages of obtaining, storing and transporting the raw material and post-use management of the packs.

## 3. Results and Discussion

### 3.1. Testing of Film Properties

#### 3.1.1. Testing the Free Shrinkage of Linear Heat-Shrinkable Film

The tested films were 0rLDPE/100LDPE (0.046 mm) and 50rLDPE/50LDPE white (0.045 mm). Table 1 shows the results that 50rLDPE/50LDPE white (0.045 mm) film had higher shrinkage in the longitudinal and transverse directions, while the differences were small in the transverse direction.

#### 3.1.2. Determination of Tear Strength (Elmendorf Method)

**Figure 3:**
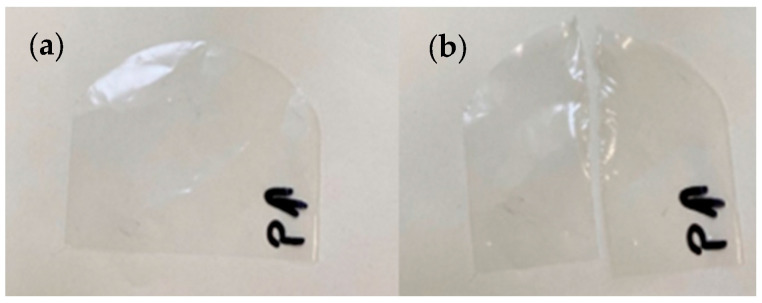
Film samples before (**a**) and after (**b**) tearing strength determination, the P marking and the arrow indicating the transversely direction of the sample example (own study).

To determine the force needed to tear the thin-film samples at a set length from the cut under specific load conditions (1600 g), an Elmendorf test was carried out in accordance with the PN-EN ISO 6383-2:2005 standard using a Pro Tear Tearing Tester, Twing machine—Albert Instrument Co. [31]. The test samples were cut in the longitudinal direction and transversely according to a template with a constant radius and a 20 mm long cut (Figure 3).

The force needed to tear each type of tested film was measured in Newtons and is shown in Table 2. In the longitudinal direction, the 50rLDPE/50LDPE color film (2.1 N) has higher tear strength than the film without the addition of recyclates, but in the transverse direction, the tear strength is lower (5.23 N).

#### 3.1.3. Comparison of Mechanical Properties of Tested Films under Static Tension

The tested films were subjected to a static tensile test under normal conditions. Previously prepared and conditioned samples (10 samples from each type of tested film) cut in the longitudinal direction and transverse direction, were stretched at a speed of 100 mm/min until they broke. The aim of these tests was to determine the basic strength characteristics of films for the thermo-shrinkable packaging of bottles. All mean values of the tensile strength, yield stress, yield stress, tensile elongation, failure stress, yield stress and sample thickness results recorded during the test, along with the standard deviation and coefficient of variation, are shown it in Table 3.

Elongation at break is a material property that informs us about the destruction of plastic during its use and processes related to the preparation of waste for reprocessing. Tests have shown that 50rLDPE/50LDPE film is characterized by lower tensile strength in the longitudinal direction of about 6 MPa and elongation at break compared with film 0rLDPE/100LDPE. The obtained results confirm our thesis of the influence of recyclates on reducing the tensile strength of the material in both directions. This is due to the presence of impurities in the 50rLDPE/50LDPE film samples, causing a notch-weakening effect [40]. The obtained results confirm the information contained in the article by Kloziński and Jakubowska [40], who indicate that with an increase in the content of recyclates in the composition of a film, the number of film inclusions increases, which consequently reduces its elongation in a static tensile test.

**Figure 4:**
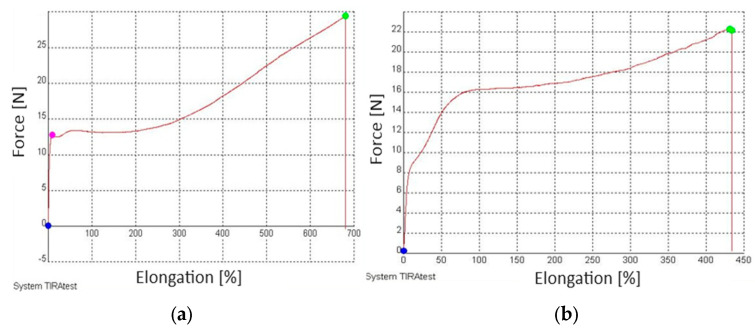
Example of a force-relative elongation diagram during a static tensile test in the Longitudinal direction: (**a**) 0rLDPE/100LDPE white film, (**b**) 50rLDPE/50LDPE white, pink point show the elongation at yield point, blue points show point for zero force and green points show forces to break the sample (own study).

A static tensile test was carried out for the 0rLDPE/100LDPE white film, for which an example strength diagram showing the relative deformation is shown in Figure 4. After exceeding the maximum value of force, the film began to stretch with a small increase in force and high elongation at the same time, until it reached an elongation of almost 700%, at which point the sample was destroyed. For 50rLDPE/50LDPE white film samples loaded in the longitudinal direction, the elongation of the samples increased proportionally to the load value for the samples, until the force value corresponding to the yield strength was reached. After exceeding the yield point, the material began to “flow”, i.e., the samples elongated with small changes in the force value. For this type of film, the average elongation at break in the longitudinal direction was 411.5% (Table 3).

Samples with the addition of recyclates achieved lower elongation values of 411–563%. The obtained results again confirm the information contained in the article by Kloziński and Jakubowska [40,41], who indicate that with an increase in the content of recyclates in the composition of the film, the number of film inclusions increases, which consequently reduces its elongation in a static tensile test.

**Figure 5:**
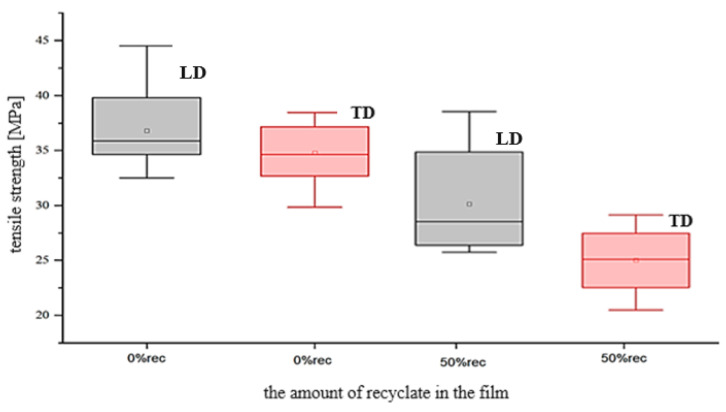
The impact of the percentage composition of recyclates in the film on their tensile strength in the longitudinal (LD) and transversal directions (TD) (own study).

Using one-way analysis of variance (ANOVA), we checked whether the percentage of recyclate in the film composition affects the tensile strength in the machine direction. The results showed that the content of recyclate in the composition of the film affects the tensile strength in the machine direction—significant statistical differences in the means were obtained (Figure 5). It was noticed that an increase in the content of recyclates in the composition of the film reduced its strength properties. Moreover, the stresses that destroyed the film sample were related to the direction of production [42]. This research has shown that in the longitudinal direction, consistent with the manufacturing process, these values were higher for both tested film samples.

#### 3.1.4. Determination of Thermal Properties

**Figure 6:**
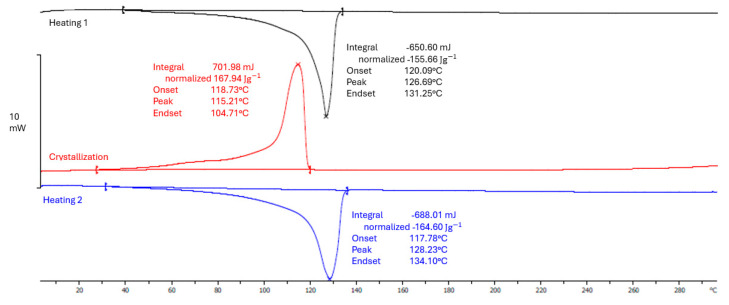
DSC curve for a sample of 0rLDPE/100LDPE white film (own study).

To check the thermal properties of the types of films analyzed in this research, differential scanning calorimetry (DSC), which is one of the basic thermoanalytical techniques, was additionally performed. During a DSC test, changes in energy (heat) occurring in the tested samples during programmed heating or cooling of the samples are recorded. The result of the experiment, called a DSC thermogram, is recorded graphically or numerically in the form of energy changes as a function of time or temperature [43]. The thermal properties of the analyzed films were determined using e Mettler Toledo apparatus according to the ISO 11357-3:2018 standard [44], in the temperature range from 30 °C to 180 °C. The samples were heated under the same conditions throughout the experiment. As a part of this research, the values of the melting point, crystallization and the degree of crystallinity of the tested film samples were obtained, which are presented in the charts below. The curves for the 0rLDPE/100LDPE white (Figure 6) film samples show a single melting point peak, with a value of 128.23 °C.

**Figure 7:**
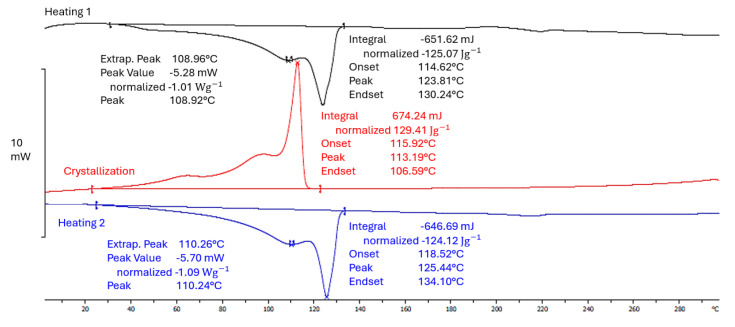
DSC curve for a sample of 50rLDPE/50LDPE white film (own study).

Meanwhile, for the 50rLDPE/50LDPE white film, the first peak was determined at a temperature of 110.24 °C and the second one at 125.44 °C (Figure 7).

The different values of melting temperatures for the analyzed film samples indicate their different thermal properties. Films containing waste material begin to melt at a lower temperature; therefore, during the mass packaging process, the temperature in the heating oven can be lowered during the film sealing stage.

### 3.2. A Comparison of the Environmental Impact of the Tested Variants of the Mass Packaging Process

To carry out this analysis, actual data on the amount of raw materials and energy used in the tested process were adopted (Table 4). The data came from measurements made in an industrial plant that deals with, among other tasks, packaging bottles in heat-shrinkable film. In both cases, the most energy-intensive stage of the packaging process was shrinking the film. In the first variant, 34.58 kWh was used in this stage, and the second, 50.85 kWh was used. In the first variant of the machine to pack bottles, 31.52 kg 0rLDPE/100LDPE film and 3.83 kg 50rLDPE/50LDPE film were used. In the second variant, 31.57 kg 50rLDPE/50LDPE film was used.

**Figure 8:**
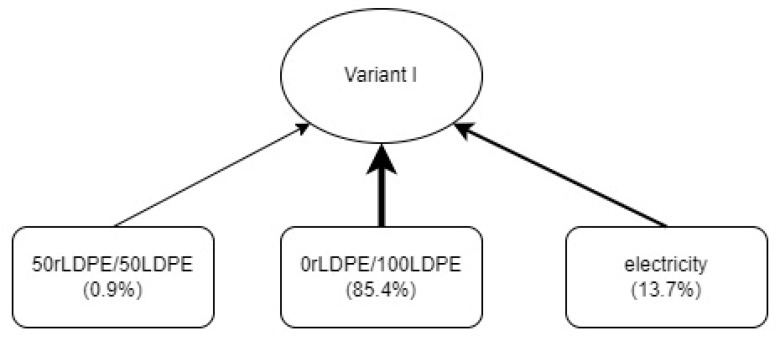
The percentage share of raw materials and media in the mass packaging process regarding the total environmental impact—variant I (own study).

As part of the analysis, raw material and media trees were created for the studied process variants. In variant I (Figure 8), the greatest potential contribution to negative impacts on the environment (85.4%) was demonstrated for the consumption of 0rLDPE/100LDPE film. Low values (0.938%) were recorded in this variant for the 50rLDPE/50LDPE packaging material. Electricity from the European energy mix in variant I was responsible for 13.7% of all negative impacts.

**Figure 9:**
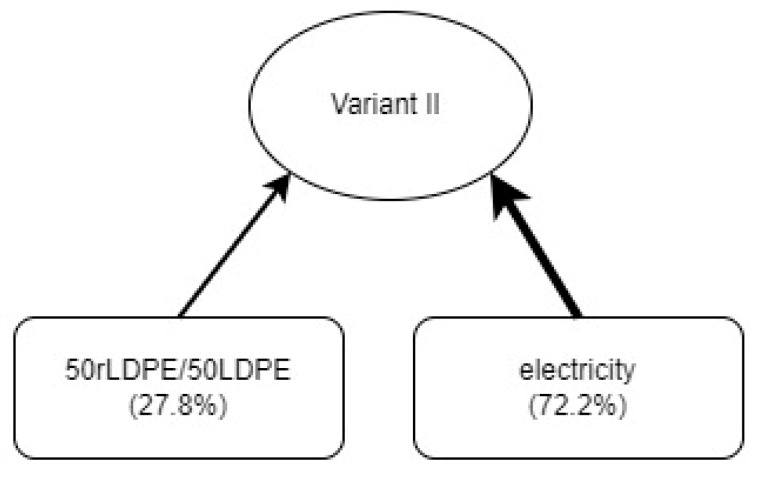
The percentage share of raw materials and media in the mass packaging process regarding the total environmental impact—variant II (own study).

In variant II (Figure 9), the dominant share of the total impact on the environment was the consumption of electricity from the European energy mix (72.2%). The remaining 27.8% of harmful impacts result from the consumption of 50rLDPE/50LDPE film.

The result of the analysis was to show the value of the total impact of individual process variants on damage to human health, ecosystems and resource use. A comparison of systems with each other was possible by using the same functional unit, system boundaries, data quality, decision rules for input and output data and impact assessment. Upon comparing the potential environmental load caused by given variants of the mass bottle packaging process to produce 1000 packs in the damage categories human health, ecosystems and resources, variant I had higher impact values (Table 5).

**Figure 10:**
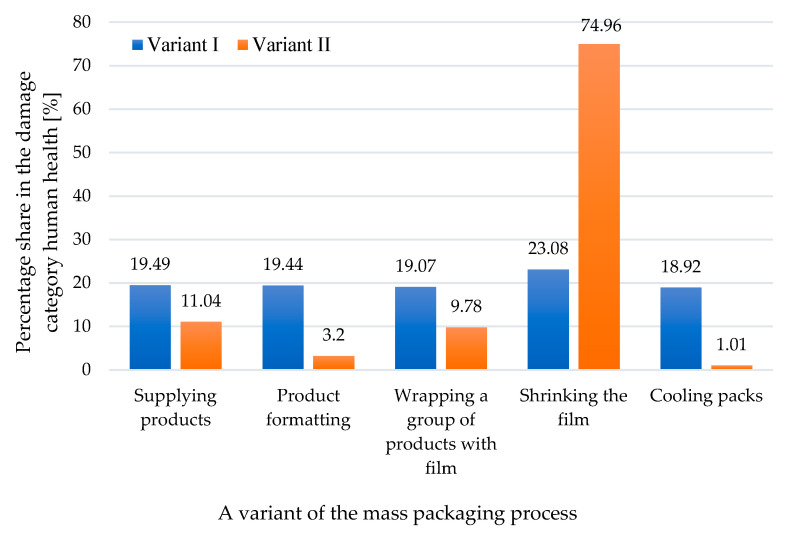
A comparison of the impact of the stages of the mass packaging process on the category of harm to human health for two tested variants (own research).

The identification of harmful effects of the mass bottle packaging process also involved verifying the impact of individual process stages on each category of damage. In the category of impact on human health, the highest share in negative impacts was the film shrinking stage in both cases (Figure 10).

**Figure 11:**
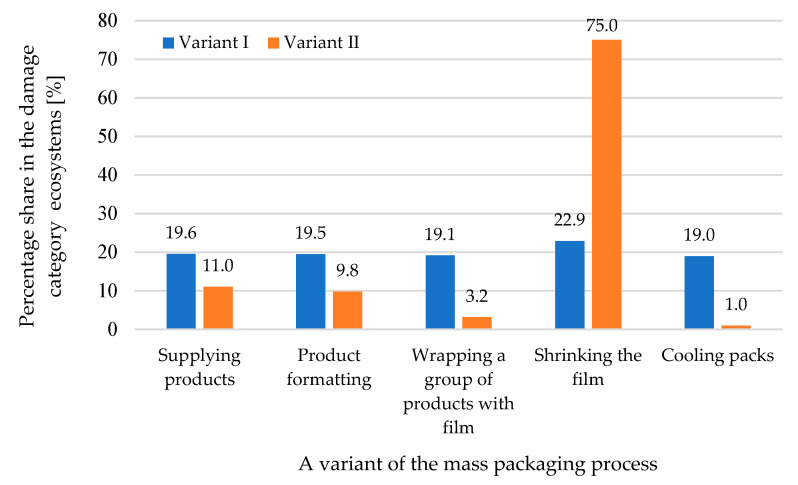
A comparison of the impact of the stages of the mass packaging process on the category of harm and damage to ecosystems for the two tested variants (own research).

Upon analyzing the results for damage to ecosystems, we noticed similar relationships to those in the previously discussed category of damage (Figure 11). For both variants, the film shrinkage stage had the greatest impact on the impact strength values in this category (22.9% and 75%).

**Figure 12:**
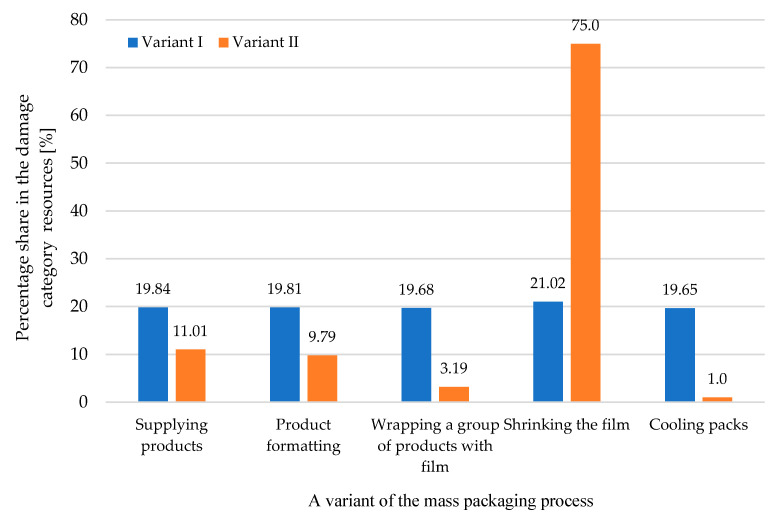
A comparison of the impact of the stages of the mass packaging process on the category of harm to resources for two tested variants (own research).

Also in the resource depletion category, the stage of film shrinkage is potentially the stage that most negatively impacts the environment (Figure 12). In the first variant, this stage covers 21.02% of potential damage in the resources category, and in the second variant of the examined process, it covers 75%.

In the three damage categories considered, the film shrinking stage has the greatest impact on the system environment (Figure 10, Figure 11 and Figure 12). This is related to the high energy consumption of this stage.

An environmental management system compliant with the PN EN ISO 14001 standard [45] has been on the market for a long time, which includes the organizational structure, procedures and activities necessary to develop, implement and maintain environmental policy. The main purpose of making such changes is to apply specific solution within companies that reduce the harmfulness of machines. The changes introduced should not disturb the company’s ability to carry out production processes and involve minimal use of natural resources and no deterioration of their quality and economics [46,47,48].

Human economic activities consume natural resources to transform them into final products. Both the processes of producing a final product and obtaining raw materials are accompanied by the formation of factors considered in terms of harmfulness and waste, which significantly burden environmental elements [49,50,51]. Present and future economic development needs to be determined and implement the right relationship between the economy and the environment. It is important to introduce modifications to production processes in the form of powering them with energy from renewable sources or using recycled materials to produce packaging.

## 4. Conclusions

The basic aim of this work was achieved by analyzing the impact of changing the type of heat-shrinkable film on the harmfulness of the bottle packaging process. This investigation covered three types of impacts that are typical of the ReCiPe 2016 (human health, ecosystems, resources). Research has shown that films available on the market with the addition of recyclates are increasingly becoming a replacement for traditional heat-shrinkable films without losing the properties necessary for the correct use of ready-made packs. Using only film with additional recyclates will also contribute to lowering the harmful impact of the bottle packaging process on the environment and the proper management of film waste. This research has shown that changing the type of film to film with the addition of recyclates results in an almost 70% reduction in the negative impact of the bottle packaging process in terms of damage to health and ecosystems, and by 85% in terms of resources. This analysis also showed that the most environmentally burdensome stage of the mass bottle packaging process is shrinking the film, which, in processes where film with the addition of recyclates is used, is responsible for over 70% of potential damage in the three categories examined. The presented modification in the process fits perfectly into the idea of a circular economy, the main goals of which focus on the rational use of resources and limiting the negative impact on the environment of manufactured products, which should, just like materials and raw materials, be used in the economy for as long as possible. Further research in the field of the processes and operation of bottle packaging machines in shrink film should be conducted in terms of modifications used in the film shrinking process, including the possible use of biodegradable products.

## Figures and Tables

**Table 1:** Test results of free shrinkage of linear heat-shrinkable film (own study).

Type of Film	Longitudinal Direction (LD)		Transverse Direction (TD)	
	Mean (%)	Standard Deviation (%)	Mean (%)	Standard Deviation (%)
0rLDPE/100LDPE (0.046 mm)	66.8	0.8	23.6	1.7
50rLDPE/50LDPE white (0.045 mm)	75.4	1.1	25.4	1.9

**Table 2:** Tear strength of tested types of film: LD—longitudinal direction; TD—transverse direction (own study).

Type of Film	Longitudinal Direction (LD), N	Transverse Direction (TD), N
0rLDPE/100LDPE (0.046 mm)	0.7	10.8
50rLDPE/50LDPE white (0.045 mm)	2.1	5.2

**Table 3:** Statistical tensile test results for the tested heat-shrinkable film samples (authors’ research).

Film Properties		0rLDPE/100LDPE (0.046 mm)		50rLDPE/50LDPE White (0.045 mm)	
		LD	TD	LD	TD
Tensile strength (MPa)	1	38.63	35.53	32.86	25.56
	2	2.78	2.38	1.66	0.81
	3	7.20	6.78	5.05	3.19
Stress at break (MPa)	1	37.62	35.10	32.49	25.47
	2	3.78	3.23	1.91	0.99
	3	10.04	9.19	5.87	3.90
Yield strength (MPa)	1	-	22.70	-	13.39
	2	-	0.64	-	0.28
	3	-	2.83	-	2.12
Elongation at maximum tensile stress (%)	1	654.84	952.62	408.8	735.61
	2	43.09	28.67	28.36	26.89
	3	6.58	3.01	6.94	3.66
Elongation at break (%)	1	655.06	953.3	411.5	736.5
	2	43.01	27.98	281.4	26.17
	3	6.57	2.93	6.84	3.55
Elongation at yield point (%)	1	9.51	7.77	-	9.88
	2	0.26	0.51	-	0.43
	3	2.70	6.53	-	4.38
Sample thickness (mm)	1	0.046	0.043	0.044	0.043
	2	0.002	0.002	0.001	0.0001
	3	4.64	3.83	3.37	1.62

**Table 4:** Inventory data on energy and film consumption for a functional unit of 1000 packs in two tested variants (own study).

Parameter	Variant I	Variant II
Electrical energy, kWh	46.10	67.81
Film, kg	35.35	37.57
Energy consumption (kWh)		
Supplying products	5.07	7.46
Product formatting	4.52	6.64
Wrapping a group of products with film	1.48	2.17
Shrinking the film	34.58	50.85
Cooling packs	0.46	0.68
Consumption of the material (kg)		
Film 0rLDPE/100LDPE	31.52	0
Film 50rLDPE/50LDPE	3.83	31.57

**Table 5:** The impact values of the tested process variants in the analyzed damage categories (own research).

Damage Category	Unit	Analyzed Process Variant	Value
Human health	DALY	I	1.77 × 10^−4^
		II	5.97 × 10^−5^
Ecosystems	species.yr	I	7.54 × 10^−7^
		II	2.39 × 10^−7^
Resources	$	I	9.77
		II	1.24

## Data Availability

The data presented in this study are available on request from the corresponding author due to privacy policies.

## References

[1] Pehnt M. (2006). Dynamic Life Cycle Assessment (LCA) of Renewable Energy Technologies. Renew. Energy.

[2] Kuzia A. (1978). System Pakowania w Folie Kurczliwe.

[3] Ragattieri A., Santarelii G. (2013). The Important Role of Packaging in Operations Management. Operations Management.

[4] Koliński A. (2011). Przegląd metod i technik oceny efektywności procesu produkcyjnego. Logistyka.

[5] Wojciechowska N. (2021). Odnawialne źródła energii jako katalizator wprowadzenia modelu gospodarki obiegu zamkniętego. Zesz. Stud. Nasze Stud..

[6] Ankiel M., Wojciechowska P., Wiszumirska K. (2021). Innowacje Opakowaniowe na Rynku Produktów Konsumpcyjnych.

[7] Yao Z., Seong H., Jang Y. (2022). Environmental toxicity and decomposition of polyethylene. Ecotoxicol. Environ. Saf..

[8] Harding K.G., Dennis J.S., Blottnitz H., Harrison S.T.L. (2007). Environmental analysis of plastic production processes: Comparing petroleum-based polypropylene and polyethylene with biologically based poly-β-hydroxybutyric acid using life cycle analysis. J. Biotechnol..

[9] Brock A., Williams I. (2020). Life cycle assessment and beverage packaging. Detritus.

[10] Martín-Lara M.A., Moreno J.A., Garcia-Garcia G., Arjandas S., Calero M. (2022). Life cycle assessment of mechanical recycling of post-consumer polyethylene flexible films based on a real case in Spain. J. Clean. Prod..

[11] Tan Q., Yang L., Wei F., Chen Y., Li J. (2023). Comparative life cycle assessment of polyethylene agricultural mulching film and alternative options including different end-of-life routes. Renew. Sustain. Energy Rev..

[12] Romero-Hernández O., Romero-Hernández S., Muñoz D., Detta-Silveira E., Palacios-Brun A., Laguna A. (2009). Environmental implications and market analysis of soft drink packaging systems in Mexico. A waste management approach. Int. J. Life Cycle Assess..

[13] Kang D.H., Auras R., Singh J. (2017). Life cycle assessment of non-alcoholic single-serve polyethylene terephthalate beverage bottles in the state of California. Resour. Conserv. Recycl..

[14] Ferrara C., Migliaro V., Ventura F., De Feo G. (2023). An economic and environmental analysis of wine packaging systems in Italy: A life cycle (LC) approach. Sci. Total Environ..

[15] Boutros M., Saba S., Manneh R. (2021). Life cycle assessment of two packaging materials for carbonated beverages (polyethylene terephthalate vs. glass): Case study for the Lebanese context and importance of the end-of-life scenarios. J. Clean. Prod..

[16] Saraiva A.B., Pacheco E.B., Gomes G.M., Visconte L.L., Bernardo C.A., Simoes C.L., Soares A.G. (2016). Comparative lifecycle assessment of mango packaging made from a polyethylene/natural fiber-composite and from cardboard material. J. Clean. Prod..

[17] Lin H., Black M.J., Walsh L., Giordano F.S., Borrion A. (2024). Life cycle assessment of baby leaf spinach: Reduction of waste through interventions in growing treatments and packaging. J. Clean. Prod..

[18] Civancik-Uslu D., Puig R., Voigt S., Walter D., Fullana-i-Palmer P. (2019). Improving the production chain with LCA and eco-design: Application to cosmetic packaging. Resour. Conserv. Recycl..

[19] Abbasi T., Jaafarzadeh Haghighi Fard N., Madadizadeh F., Eslami H., Ebrahimi A.A. (2023). Environmental Impact Assessment of Low-Density Polyethylene and Polyethylene Terephthalate Containers Using a Life Cycle Assessment Technique. J. Polym. Environ..

[20] Dong H., Yang G., Zhang Y., Yang Y., Wang D., Zhou C. (2022). Recycling, disposal, or biodegradable-alternative of polyethylene plastic film for agricultural mulching? A life cycle analysis of their environmental impacts. J. Clean. Prod..

[21] Horodytska O., Kiritsis D., Fullana A. (2020). Upcycling of printed plastic films: LCA analysis and effects on the circular economy. J. Clean. Prod..

[22] Choi B., Yoo S., Park S.I. (2018). Carbon footprint of packaging films made from LDPE, PLA, and PLA/PBAT blends in South Korea. Sustainability.

[23] Salwa H.N., Sapuan S.M., Mastura M.T., Zuhri M.Y.M., Ilyas R.A. (2021). Life Cycle Assessment of Bio-Based Packaging Products. Bio-Based Packaging: Material, Environmental and Economic Aspects.

[24] Bala A., Arfelis S., Oliver-Ortega H., Méndez J.A. (2022). Life cycle assessment of PE and PP multi film compared with PLA and PLA reinforced with nanoclays film. J. Clean. Prod..

[25] Tunçok-Çeşme B., Yıldız-Geyhan E., Çiftçioğlu G.A. (2024). Environmental Life Cycle Assessment of Two Types of Flexible Plastic Packaging under a Sustainable Circular Economy Approach. Sustainability.

[26] Drabczyk M., Kruczek B. (2016). Wpływ dodatku materiału nieprzetwarzalnego na wytrzymałość na rozciąganie folii LDPE, Problemy Nauk Stosowanych. Probl. Nauk. Stosowanych..

[27] Rojek M., Szymiczek M. (2014). Ocena wpływu dodatku regranulatu na własności opakowaniowej folii pęcherzykowej. Przetwórstwo Tworzyw..

[28] (2020). Tworzywa Sztuczne—Oznaczanie Właściwości Mechanicznych Przy Statycznym Rozciąganiu—Część 1: Zasady Ogólne.

[29] (2019). Tworzywa Sztuczne—Oznaczanie Właściwości Przy Rozciąganiu—Część 3: Warunki Badań Folii i Płyt.

[30] (2020). Standard Test Method for Unrestrained Linear Thermal Shrinkage of Plastic Film and Sheeting.

[31] (2005). Tworzywa Sztuczne—Folie i Płyty—Oznaczanie Wytrzymałości na Rozdzieranie—Część 2: Metoda Elmendorfa.

[32] Pauer E., Tacker M., Gabriel V., Krauter V. (2020). Sustainability of flexible multilayer packaging: Environmental impacts and recyclability of packaging for bacon in block. Clean. Environ. Syst..

[33] Sazdovski A., Bala A., Palmer P. (2021). Linking LCA literature with circular economy value creation: A review on beverage packaging. Sci. Total Environ..

[34] Bałdowska-Witos P., Kruszelnicka W., Kasner R., Piotrowska K., Tomporowski A., Rudnicki J., Opielak M., Flizikowski J. (2019). Assessment of the Environmental Impact of a Car Tire throughout Its Lifecycle Using the LCA Method. Materials.

[35] Idzikowski A., Walichnowska P. (2022). The management of the technological process of a product on the example a shrink film in the aspect life cycle assessment. Syst. Saf. Hum.—Tech. Facil.—Environ..

[36] Janicka J., Koźmińska R. (2009). Tekstylia w materiałach kompozytowych. Tech. Wyr. Włókiennicze.

[37] Walichnowska P., Idzikowski A. (2023). Assessment and Analysis of the Environmental Impact of the Thermo-Shrinkable Packaging Process on the Way the Packaging Machine is Powered Based on LCA. Manag. Syst. Prod. Eng..

[38] Leda P., Idzikowski A., Piasecka I., Bałdowska-Witos P., Cierlicki T., Zawada M. (2023). Management of Environmental Life Cycle Impact Assessment of a Photovoltaic Power Plant on the Atmosphere, Water, and Soil Environment. Energies.

[39] Pieragostini C., Mussati M.C., Pío Aguirre P. (2012). On process optimization considering LCA methodology. J. Environ. Manag..

[40] Kloziński A., Jakubowska P. (2010). Folie wytwarzane z dodatkiem recyklatów—Przetwórstwo, właściwości. Inż. Apar. Chem..

[41] Kloziński A., Jakubowska P. (2012). Właściwości mechaniczne folii poużytkowych stosowanych w rolnictwie. Inż. Apar. Chem..

[42] Bembenek M., Kowalski Ł., Kosoń-Schab A. (2022). Research on the Influence of Processing Parameters on the Specific Tensile Strength of FDM Additive Manufactured PET-G and PLA Materials. Polymers.

[43] Kowalska D. (2017). Różnicowa kalorymetria skaningowa DSC, ciśnieniowa różnicowa kalorymetria skaningowa PDSC, StepScan DSC, temperaturowo modulowana DSC szybka i superszybka DSC w badaniu żywności. Apar. Badaw. Dydakt..

[44] (2018). International Organization for Standardization. Plastics—Differential Scanning Calorimetry (DSC)—Part 3: Determination of Temperature and Enthalpy of Melting and Crystallization.

[45] (2005). The Polish Committee for Standardization. Environmental Management Systems—Requirements and Guidelines for Use.

[46] Qin Y., Cucurachi S., Suh S. (2020). Perceived Uncertainties of Characterization in LCA: A Survey. Int. J. Life Cycle Assess..

[47] Ram A., Sharma P. (2017). A study on Life Cycle Assessment. Int. J. Eng. Adv. Technol..

[48] Wikström F., Williams H., Vergheseb K., Clunec S. (2014). The influence of packaging attributes on consumer behaviour in food-packaging life cycle assessment studies—A neglected topic. J. Clean. Prod..

[49] Williams H., Wikström F. (2011). Environmental impact of packaging and food losses in a life cycle perspective: A comparative analysis of five food items. J. Clean. Prod..

[50] Górzyński J. (2007). Podstawy Analizy Środowiskowej Wyrobów i Obiektu.

[51] Zhao S., You F. (2019). Comparative Life-Cycle Assessment of Li-Ion Batteries through Process-Based and Integrated Hybrid Approaches. ACS Sustain. Chem. Eng..

